# ADP-ribosyltransferase PARP11 suppresses Zika virus in synergy with PARP12

**DOI:** 10.1186/s13578-021-00628-y

**Published:** 2021-06-29

**Authors:** Lili Li, Yueyue Shi, Sirui Li, Junxiao Liu, Shulong Zu, Xin Xu, Meiling Gao, Nina Sun, Chaohu Pan, Linan Peng, Heng Yang, Genhong Cheng

**Affiliations:** 1 Center for Systems Medicine, Institute of Basic Medical Sciences, Chinese Academy of Medical Sciences and Peking Union Medical College, Beijing, 100005 China; 2 Suzhou Institute of Systems Medicine, Suzhou, 215123 Jiangsu China; 3 Lineberger Comprehensive Cancer Center, University of North Carolina At Chapel Hill, Chapel Hill, NC 27599 USA; 4 CAS Key Laboratory of Infection and Immunity, Institute of Biophysics, Chinese Academy of Sciences, Chaoyang District, Beijing, 100101 China; 5 Department of Microbiology, Immunology and Molecular Genetics, University of California, Los Angeles, CA 90095 USA

**Keywords:** ADP-ribosyltransferase, PARP11, PARP12, Zika virus, NS1 and NS3, Anti-viral ISGs

## Abstract

**Background:**

Zika virus (ZIKV) infection and ZIKV epidemic have been continuously spreading silently throughout the world and its associated microcephaly and other serious congenital neurological complications poses a significant global threat to public health. Type I interferon response to ZIKV infection in host cells suppresses viral replication by inducing the expression of interferon-stimulated genes (ISGs).

**Methods:**

The study aims to demonstrate the anti-ZIKV mechanism of PARP11. PARP11 knock out and overexpressing A549 cell lines were constructed to evaluate the anti-ZIKV function of PARP11. *PARP11*^*−/−*^, *PARP12*^*−/−*^ and *PARP11*^*−/−*^*PARP12*^*−/−*^ HEK293T cell lines were constructed to explain the synergistic effect of PARP11 and PARP12 on NS1 and NS3 protein degradation. Western blotting, immunofluorescence and immunoprecipitation assay were performed to illustrate the interaction between PARP11 and PARP12.

**Results:**

Both mRNA and protein levels of PARP11 were induced in WT but not *IFNAR1*^*−/−*^ cells in response to IFNα or IFNβ stimulation and ZIKV infection. ZIKV replication was suppressed in cells expressed PARP11 but was enhanced in *PARP11*^*−/−*^ cells. PARP11 suppressed ZIKV independently on itself PARP enzyme activity. PARP11 interacted with PARP12 and promoted PARP12-mediated ZIKV NS1 and NS3 protein degradation.

**Conclusion:**

We identified ADP-ribosyltransferase *PARP11* as an anti-ZIKV ISG and found that it cooperated with PARP12 to enhance ZIKV NS1 and NS3 protein degradation. Our findings have broadened the understanding of the anti-viral function of ADP-ribosyltransferase family members, and provided potential therapeutic targets against viral ZIKV infection.

**Supplementary Information:**

The online version contains supplementary material available at 10.1186/s13578-021-00628-y.

## Introduction

Zika virus (ZIKV) was first isolated in 1947 in the Zika forest of Uganda from an infected rhesus macaque [1]. Since its discovery, ZIKV stayed relatively silent for almost 70 years until recent outbreaks in Pacific Islands and Brazil. By December 2015, 18 states of Brazil had reported autochthonous ZIKV transmission and large numbers of cases of infection and its associated diseases such as microcephaly were reported in 2015 and 2016 [2–4]. On February 1, 2016, the World Health Organization (WHO) declared ZIKV outbreak and its associated clinical manifestations as a Public Health Emergency of International Concern (PHEIC). ZIKV continues to develop and spread silently throughout the world in the form of asymptomatic infections. During September–November 2018, the biggest Indian break was reported from Rajasthan and Madhya Pradesh states of India. Up to July 2019, 87 countries reported ZIKV transmission including 1,274,974 diagnosed cases in Brazil [5]. Recently, an Africa strain ZIKV infection was found in Brazil in addition to the prevalent Asia strain, suggesting more attention should be paid to another outbreak of ZIKV epidemic [6].

ZIKV infection is asymptomatic in up to 80% adults, the remaining 20% infected adults are characterized by low fever, arthralgia, maculopapular rash accompanied by pruritis, and conjunctivitis. Moreover, ZIKV infection in adults was associated with Guillain–Barre syndrome and infection during pregnancy can cause infants’ microcephaly, intrauterine growth restriction and other birth defects [7–9]. Thousands of increased cases of fetal abnormalities, including microcephaly, were reported up to February 2016 in ZIKV infected areas [9–11]. At present, vaccines or antivirals to treat ZIKV infection are unavailable [12–14]. Thereby, a comprehensive research for anti-ZIKV genes and more potential therapy targets are urgently needed to be identified.

Type I interferon (IFN-I) and interferon-stimulated genes (ISGs) constitute the vital part of innate immune system against virus infection in vertebrate. Viral infection induces the production of IFN-I and about 300 ISGs which exert a broad-spectrum anti-viral effect. The potential of IFN-I and ISGs against ZIKV infection has been indicated [15–17]. Our previously work also identified *CH25H* and *PARP12* as critical anti-ZIKV ISGs, which suppressed ZIKV infection and replication [18, 19]. However, additional ISGs are likely involved in controlling ZIKV replication. The family of poly-adenosine 5’-diphosphate (ADP)-ribose polymerases (PARPs), also known as ADP-ribosyltransferases, mediates a unique translational modification called ADP-ribosylation by transferring of ADP-ribose from nicotinamide adenine dinucleotide (NAD^+^) to target proteins [20–22]. Among the 17 PARPs expressed in human cells, several PARPs, such as PARP13, PARP9, PARP10, PARP14, PARP12 and PARP5, have been identified as ISGs and are involved in anti-viral response [23–25]. In an anti-ZIKV ISG screening, we reported mono ADP-ribosyltransferase PARP11 with anti-ZIKV function but without detailed mechanism described [18]. On the other hand, Guo et al. reported that PARP11 promotes vesicular stomatitis virus (VSV) and herpes simplex virus-1 (HSV-1) infection by inhibiting the interferon response [26]. These results indicate a complex involvement of PARP11 in different viral infection.

In this work, we found that PARP11 was up-regulated in response to IFN-I stimulation and ZIKV infection, and acted as an anti-ZIKV ISG. Unlike PARP12, which suppressed ZIKV replication through PARP enzymatic activity dependent degradation of ZIKV NS1 and NS3 proteins, PARP11 suppressed ZIKV replication independent on its PARP enzymatic activity. Instead, PARP11 interacted and cooperated with PARP12 in suppressing ZIKV replication, which provided a new insight on understanding the mechanisms responsible for different PARP family members in viral infection.

## Results

### The expression of PARP11 can be induced by IFN and ZIKV infection

**Fig.1:**
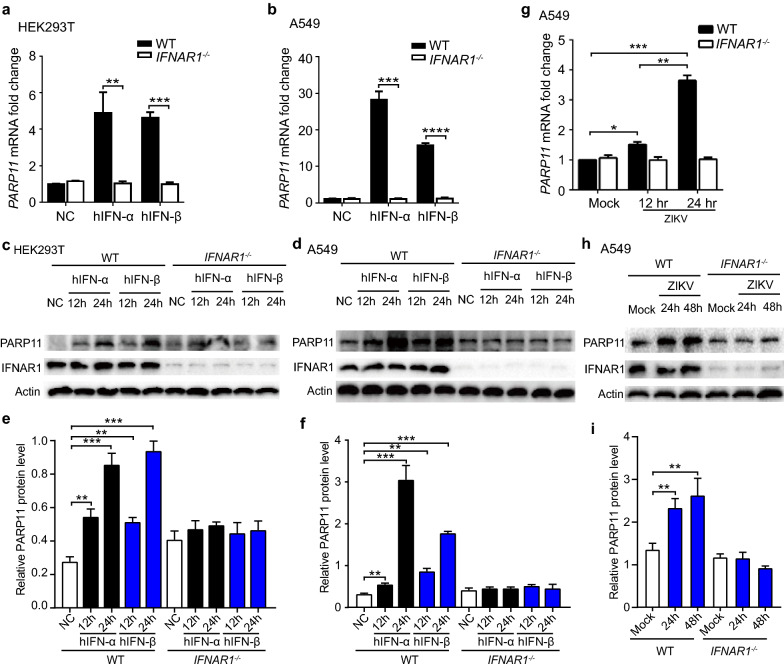
The expression of PARP11 can be induced by IFN and ZIKV infection. **a**, **b** qRT-PCR analysis of *PARP11* expression in WT and *IFNAR1*^*−/−*^ HEK293T (**a**) or A549 (**b**) cells treated with recombinant human IFN-α (1000 U/mL), IFN-β (20 ng/mL) and control for 24 h. **c**, **d** Western blotting analysis of *PARP11* expression in WT and *IFNAR1*^*−/−*^ HEK293T (**c**) or A549 (**d**) cells treated with recombinant human IFN-α (1000 U/mL), IFN-β (20 ng/mL) and control for 12 and 24 h. **e** Densitometry analysis of the data in (**c**). **f** Densitometry analysis of the data in (**d**). **g** qRT-PCR analysis of *PARP11* expression in WT and *IFNAR1*^*−/−*^ A549 cells infected with ZIKV for the indicated times. **h** Western blotting analysis of *PARP11* expression in WT and *IFNAR1*^*−/−*^ A549 cells infected with ZIKV for the indicated times. **i** Densitometry analysis of the data in (**h**). qRT-PCR (**a**, **b** and **g**) and densitometry (**e**, **f** and **i**) data are mean ± SEM from three independent experiments. Western blotting results (**c**, **d** and **h**) are representative images of three independent experiments. *P < 0.05, **P < 0.01, ***P < 0.001 and ****P < 0.0001 by Student’s *t* test

In our previously screening for ISGs with activity against ZIKV, we have identified PARP12 that suppresses ZIKV through PARP-dependent degradation of NS1 and NS3 viral proteins [18]. We also noticed that PARP11 showed anti-ZIKV activity. PARP11 was reported as an ISG [26]. To characterize the induction of PARP11 by IFN-I, we stimulated WT and IFNα/β receptor subunit 1 (IFNAR1)-deficient HEK293T and A549 cells with human IFN-I (IFN-α and IFN-β) and quantified the mRNA and protein levels of PARP11 by quantitative real-time PCR (qRT-PCR) and western blotting assay. We found that the PARP11 mRNA and protein levels were induced by IFN-I in WT HEK293T and A549 cells but not in the corresponding *IFNAR1*^*−/−*^ cells (Fig. 1a–f). Similar to exogenous IFN treatment, ZIKV infection also induced PARP11 mRNA and protein expressions in WT but not in *IFNAR1*^*−/−*^ A549 cells (Fig. 1g–i). These results indicate that *PARP11* is an ISG induced by IFN-I and ZIKV infection.

### PARP11 suppresses ZIKV replication in vitro

**Fig. 2:**
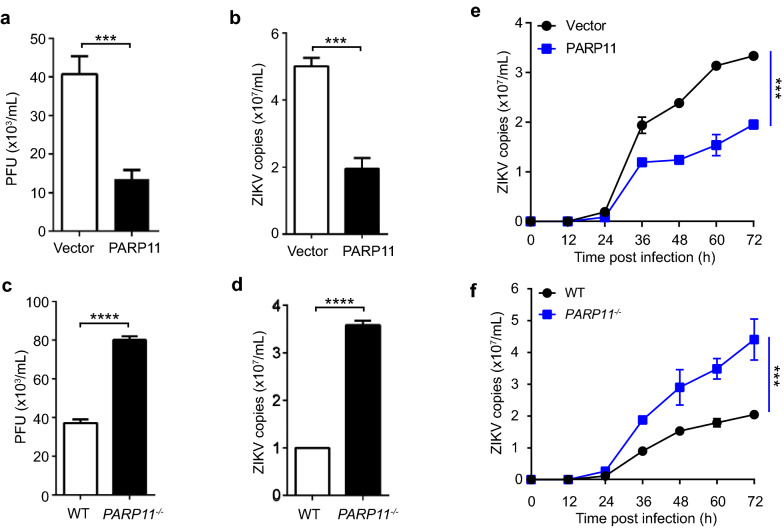
PARP11 suppresses ZIKV replication in vitro. **a, b** PARP11-overexpressing or control vector-transfected A549 cells were infected with ZIKV. Viral accumulation after 48 h in the culture supernatants (**a**) and cell lysates (**b**) were measured by plaque assay (**a**) or qRT-PCR (**b**). PFU, plaque-forming unites. **c**, **d** WT or *PARP11*^*−/−*^ A549 cells were infected with ZIKV. Viral accumulation after 48 h in the culture supernatants (**c**) and cell lysates (**d**) were measured by plaque assay (**c**) or qRT-PCR (**d**). **e**, **f** Growth curve of ZIKV in PARP11-overexpressing or control vector-transfected A549 cells (**e**) and WT or *PARP11*^*−/−*^ A549 cells (**f**). qRT-PCR and viral titers data (**a**–**f**) are mean ± SEM from three independent experiments. ***P < 0.001 and ****P < 0.0001 by Student’s *t* test

To further identify the anti-ZIKV activity, we generated both *PARP11*-knockout (Additional file 1: Fig. S1a–c) and PARP11-overexpressing (Additional file 1: Fig. S1d) A549 cell lines. We found that ZIKV replication was suppressed in PARP11-overexpressing A549 cells as compared with the parental WT A549 cells (Fig. 2a, b). Conversely, ZIKV replication was enhanced in *PARP11*^−/−^ cells (Fig. 2c, d). Furthermore, we monitored the growth kinetics of ZIKV in vector control, PARP11-overexpressing, WT and *PARP11*^*−/−*^ A549 cells. The results also showed that the ZIKV replication rate was decreased in PARP11-overexpressing cells and was increased in *PARP11*^*−/−*^ cells (Fig. 2e, f). Therefore, *PARP11* is an anti-ZIKV ISG that may play an important role in innate immune defense against ZIKV replication.

### PARP11 suppresses ZIKV independent on the regulation of IFNAR1 protein level

A recent report indicated that PARP11 inhibited the interferon response by reducing the protein level of IFNAR1 [26] to promote VSV and HSV-1 replication.

**Fig. 3:**
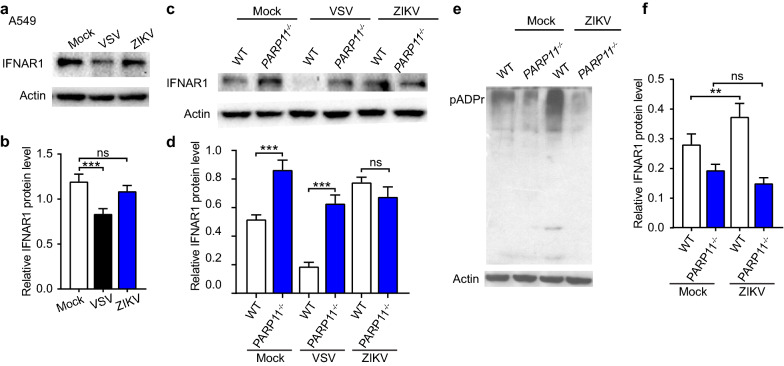
PARP11 suppresses ZIKV independent on the regulation of IFNAR1 protein level. **a** Western blotting analysis of lysates from A549 cells infected with VSV, ZIKV and mock control. **b** Densitometry analysis of the data in (**a**). **c** Western blotting analysis of lysates from WT and *PARP11*^*−/−*^ A549 cells infected with VSV, ZIKV and mock control. **d** Densitometry analysis of the data in (**c**). **e** Western blotting analysis of lysates from WT and *PARP11*^*−/−*^ A549 cells infected with ZIKV and mock control. **f** Densitometry analysis of the data in (**e**). Densitometry analysis data (**b**, **d** and **f**) are mean ± SEM pooled from three independent experiments. Western blotting results (**a**, **c** and **e**) are presentative of three independent experiments. ns, not significant, **P < 0.01, and ***P < 0.001 by Student’s *t* test

In our screening and validation results, however, we found that PARP11 suppressed ZIKV replication (Fig. 2a–f). To test whether PARP11 suppressed ZIKV by regulating protein level of IFNAR1, we infected WT A549 cells with VSV and ZIKV and detected protein level of IFNAR1 by Western blotting. The result showed that ZIKV infection did not obviously change IFNAR1 protein level, while VSV infection decreased IFNAR1 protein level significantly (Fig. 3a, b). We also compared the protein levels of IFNAR1 in WT and *PARP11*^*−/−*^ A549 cells infected with VSV and ZIKV. While significant IFNAR1 downregulation was observed in WT and *PARP11*^*−/−*^ A549 cells in response to VSV infection, the IFNAR1 protein level showed no obviously change in both WT and *PARP11*^*−/−*^ A549 cells upon ZIKV infection (Fig. 3c, d). On the other hand, we detected a strong increase in protein ADP-ribosylation in WT but not *PARP11*^*−/−*^ cells upon ZIKV infection (Fig. 3e, f). These results indicate that PARP11 may suppress ZIKV replication by inducing either host or viral protein ADP-ribosylation in ZIKV infected cells instead of regulating the IFNAR1 protein level.

### PARP11 suppresses ZIKV independent on its PARP enzyme activity

**Fig. 4:**
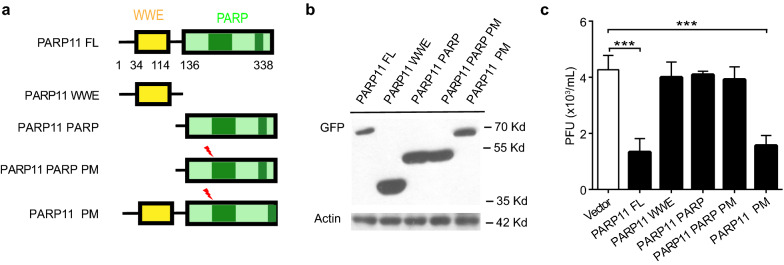
PARP11 suppresses ZIKV independent on its PARP enzyme activity. **a** Sketch map of the functional regions of full length (FL) PARP11 and mutant constructs. FL: full length; WWE: WWE domain; PARP: Poly (ADP-ribose) polymerase domain. PM: Point mutation to inactivate PARP enzyme activity. **b** Western blotting analysis of lysates from HEK293T cells transfected with the plasmids encoding GFP-tagged FL and mutant PARP11 constructs. **c** HeLa cells were transfected with FL PARP11 and mutant constructs and infected with ZIKV 24 h after transfection. 48 h post infection, ZIKV titers in HeLa cells culture supernatant were measured by plaque assay. Western blotting results (**b**) are presentative of three independent experiments. Viral titers (**c**) are mean ± SEM from three independent experiments. ***P < 0.001 by Student’s *t* test

PARP11 contains WWE domain at the N terminus and PARP domain at the C terminus. The WWE domain is a common interaction module that participates in both ubiquitination and ADP-ribosylation [27]. The PARP domain contains the PARP enzyme activity, which mediates the posttranslational modification of target proteins [28, 29]. The Gly^205^ within the PARP domain of human PARP11 binds the amide group of NAD^+^, which is essential for PARP enzyme activity. To identify whether the PARP enzyme activity of PARP11 is responsible for the anti-ZIKV function, we constructed PARP11 deletion and PARP enzyme activity lost (PARP11 G205A) mutants (Fig. 4a). The expression and expected protein size of these mutants were verified by Western blotting (Fig. 4b). HeLa cells were transfected with these mutants and subsequently infected by ZIKV to determine how the different domains and PARP enzyme activity affected viral infection. Full-length WT or enzyme inactive PARP11 strongly suppressed ZIKV replication, whereas PARP11 deletion mutants lacking the WWE domain or the PARP domain showed no suppression on ZIKV replication (Fig. 4c). These results indicate that PARP11 suppresses ZIKV replication independent on its PARP enzyme activity.

### PARP11 promotes PARP12-mediated ZIKV NS1 and NS3 protein degradation

**Fig. 5:**
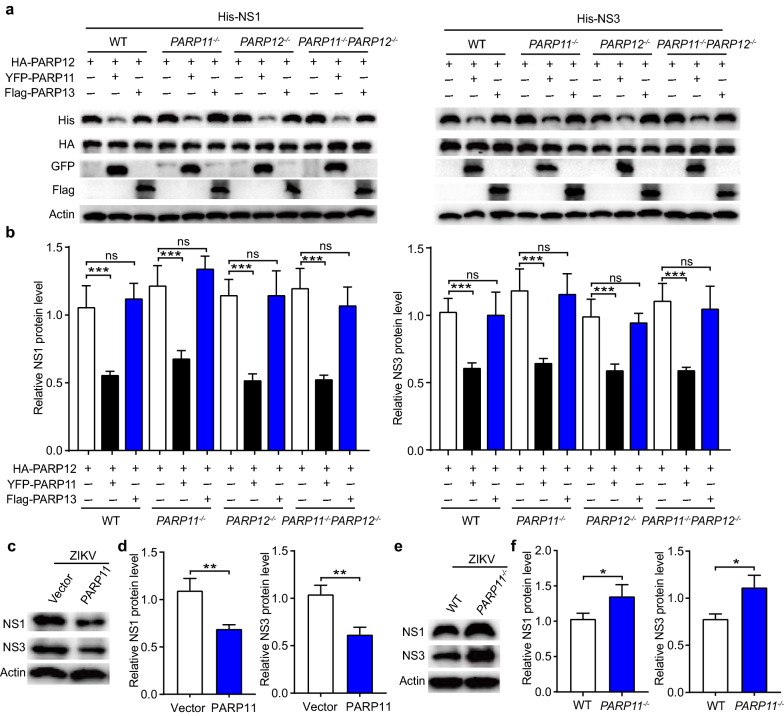
PARP11 promotes PARP12-mediated ZIKV NS1 and NS3 protein degradation. **a** Western blotting analysis of cell lysates from WT, *PARP11*^*−/−*^, *PARP12*^*−/−*^ and *PARP11*^*−/−*^*PARP12*^*−/−*^ HEK293T transfected with HA-PARP12, YFP-PARP11, Flag-PARP13 and His-NS1 or His-NS3 plasmids as indicated. **b** Densitometry analysis of the data in (**a**). **c** Western blotting analysis of expression of NS1 and NS3 proteins from PARP11-overexpressing or control vector-transfected A549 cells infected with ZIKV for 48 h. **d** Densitometry analysis of the data in (**c**). **e** Western blotting analysis of expression of NS1 and NS3 proteins from WT or *PARP11*^*−/−*^ A549 cells infected with ZIKV for 48 h. **f** Densitometry analysis of the data in (**e**). Western blotting results (**a**, **c** and **e**) are presentative of three independent experiments. Densitometry analysis data (**b**, **d** and **f**) are mean ± SEM pooled from three independent experiments. ns, not significant, *P < 0.05, **P < 0.01, and ***P < 0.001 by Student’s *t* test

In our previous work, we identified PARP12 suppressed ZIKV through PARP-dependent degradation of NS1 and NS3 viral protein. The PARP enzyme activity of PARP12 is essential for ZIKV suppression. However, in this work, we found that PARP11 suppressed ZIKV independent on its PARP enzyme activity but can regulate protein ADP-ribosylation in ZIKV infected host cells. To test the relationship between PARP11 and PARP12, we co-transfected His-NS1 and NS3 with HA-PARP12, YFP-PARP11 or Flag-PARP13 as control into WT, *PARP11*^*−/−*^, *PARP12*^*−/−*^ and *PARP11*^*−/−*^*PARP12*^*−/−*^ HEK293T cells. Compared to PARP12 co-transfected with vector group, PARP12 co-transfection with PARP11 but not PARP13 further reduced the abundance of NS1 and NS3 proteins (Fig. 5a, b). We also detected the impacts of PARP11 on the ZIKV-encoded NS1 and NS3 protein levels in ZIKV infected cells and found that the endogenous NS1 and NS3 protein levels were decreased in PARP11-overexpressed cells and were increased in *PARP11*^*−/−*^ cells (Fig. 5c–f). These results indicates that PARP11 can cooperate with PARP12 in -mediating ZIKV NS1 and NS3 degradation and may play an important role in controlling the levels of NS1 and NS3 in ZIKV infected cells.

### PARP11 promotes PARP12-mediated NS1 and NS3 degradation independent on its PARP enzyme activity

**Fig. 6:**
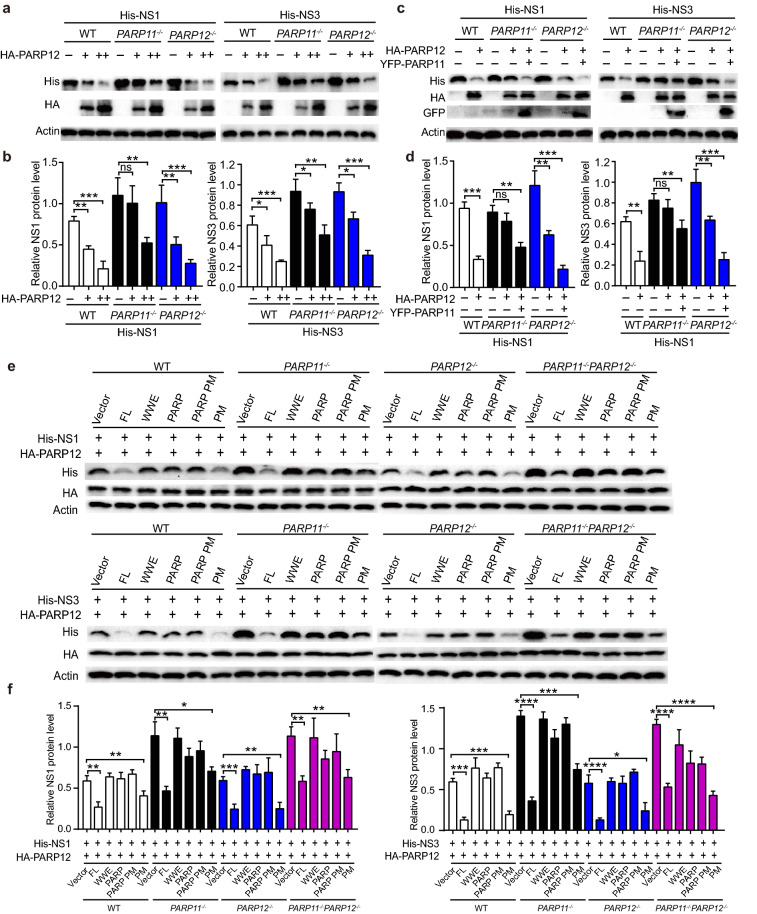
PARP11 promotes PARP12-mediated NS1 and NS3 degradation independent on its PARP enzyme activity. **a** Western blotting analysis of cell lysates from WT, *PARP11*^*−/−*^ and *PARP12*^*−/−*^ HEK293T transfected with His-NS1 or NS3 and increasing amounts of HA-PARP12 plasmids (0, 250, 500 ng). **b** Densitometry analysis of the data in (**a**). **c** Western blotting analysis of cell lysates from WT, *PARP11*^*−/−*^ and *PARP12*^*−/−*^ HEK293T transfected with HA-PARP12, YFP-PARP11 and His-NS1 or His-NS3 plasmids. **d** Densitometry analysis of the data in (**c**). **e** Western blotting analysis of cell lysates from WT, *PARP11*^*−/−*^, *PARP12*^*−/−*^ and *PARP11*^*−/−*^*PARP12*^*−/−*^ HEK293T transfected with HA-PARP12, His-NS1/NS3 and FL PARP11 and mutant constructs plasmids. FL: full length; WWE: WWE domain; PARP: Poly (ADP-ribose) polymerase domain. PARP PM: PARP domain with point mutation to inactivate PARP enzyme activity. PM: PARP11 full length protein with point mutation to inactivate PARP enzyme activity. **f** Densitometry analysis of the data in (**e**). Western blotting results (**a**, **c** and **e**) are presentative of three independent experiments. Densitometry analysis data (**b**, **d** and **f**) are mean ± SEM pooled from three independent experiments. ns, not significant, *P < 0.05, **P < 0.01, ***P < 0.001, and ***P < 0.0001 by Student’s *t* test.

To further confirm the involvement of PARP11 in PARP12-mediated NS1 and NS3 degradation, we co-transfected His-NS1 or NS3 with increasing amount of HA-PARP12 plasmids in WT, *PARP11*^*−/−*^, and *PARP12*^*−/−*^ HEK293T cells. Compared to WT HEK293T cells, PARP12 showed weaker ability to degrade NS1 and NS3 in *PARP11*^*−/−*^ HEK293T cells (Fig. 6a, b). When PARP11 was transfected back, NS1 and NS3 degradations mediated by PARP12 were increased (Fig. 6c, d). We also examined the PAPR11 mutants on NS1 and NS3 degradation when co-transfected with HA-PARP12 into WT, *PARP11*^*−/−*^, *PARP12*^*−/−*^ and *PARP11*^*−/−*^*PARP12*^*−/−*^ HEK293T cells. Only the full-length WT and PARP enzyme lost mutation enhanced NS1 and NS3 degradation in synergy with PARP12, PARP11 deletion mutants lacking the WWE domain or the PARP domain did not show impact on enhancing PARP12-mediated NS1 and NS3 degradation (Fig. 6e, f). Interestingly, we observed that PARP11 enzyme lost mutant degraded NS1 and NS3 less efficient than WT PARP11 in *PARP11*^*−/−*^ HEK293T cells (Fig. 6e, f). Together, these results show that PARP11 promotes the degradation of ZIKV NS1 and NS3 proteins in synergy with PARP12.

### PARP11 suppresses ZIKV replication mostly dependent on PARP12

**Fig. 7:**
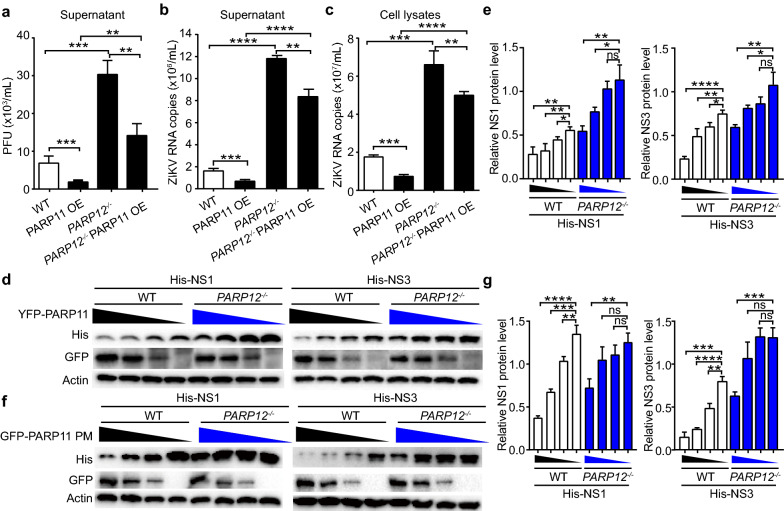
PARP11 suppresses ZIKV mostly dependent on PARP12. **a**–**c** PARP11-overexpressing or control vector-transfected WT and *PARP12*^*−/−*^ A549 cells were infected with ZIKV. Viral accumulation after 48 h in the culture supernatants (**a** and **b**) and cell lysates (**c**) were measured by plaque assay (**a**) or qRT-PCR (**b** and **c**). PFU, plaque-forming unites. **d** Western blotting analysis of cell lysates of cell lysates from WT and *PARP12*^*−/−*^ HEK293T cells transfected with His-NS1 or NS3 and decreasing amounts of YFP-PARP11 plasmids (500, 300, 100, 0 ng). **e** Densitometry analysis of the data in (**d**). **f** Western blotting analysis of cell lysates of cell lysates from WT and *PARP12*^*−/−*^ HEK293T cells transfected with His-NS1 or NS3 and decreasing amounts of YFP-PARP11 enzyme lost mutant plasmids (500, 300, 100, 0 ng). **g** Densitometry analysis of the data in (**f**). qRT-PCR, viral titers and densitometry analysis data (**a**–**c, e** and **g**) are mean ± SEM from three independent experiments. Western blotting results (**d** and **f**) are presentative of three independent experiments. **P < 0.01, ***P < 0.001 and ****P < 0.0001 by Student’s *t* test

To further determine whether PARP11 suppressed ZIKV dependent upon PARP12, we compared the replication of ZIKV in WT and *PARP12*^*−/−*^ A549 cells in the presence or absence of PARP11 over expression. The results showed that although ZIKV replication was reduced in both WT and *PARP12*^*−/−*^ A549 cells upon PARP11 overexpression PARP11 seemed to be more effective in inhibiting ZIKV replication in *PARP12*^*−/−*^ A549 cells as compared with WT A549 cells (Fig. 7a–c). We then checked whether the degradations of NS1 and NS3 mediated by PARP11 WT and PARP11 enzyme lost mutant were also impacted by PARP12. Western blot analysis revealed that PARP11 WT and PARP11 enzyme lost mutant can still degrade NS1 and NS3 in *PARP12*^*−/−*^HEK293T cells but at an efficiency significantly lower than that in WT HEK293T cells (Fig. 7d–g). These results suggest that PARP11 suppresses NS1 and NS3 degradation and ZIKV replication mostly dependent on the existence of PARP12.

### PARP11 interacts and co-localizes with PARP12

**Fig. 8:**
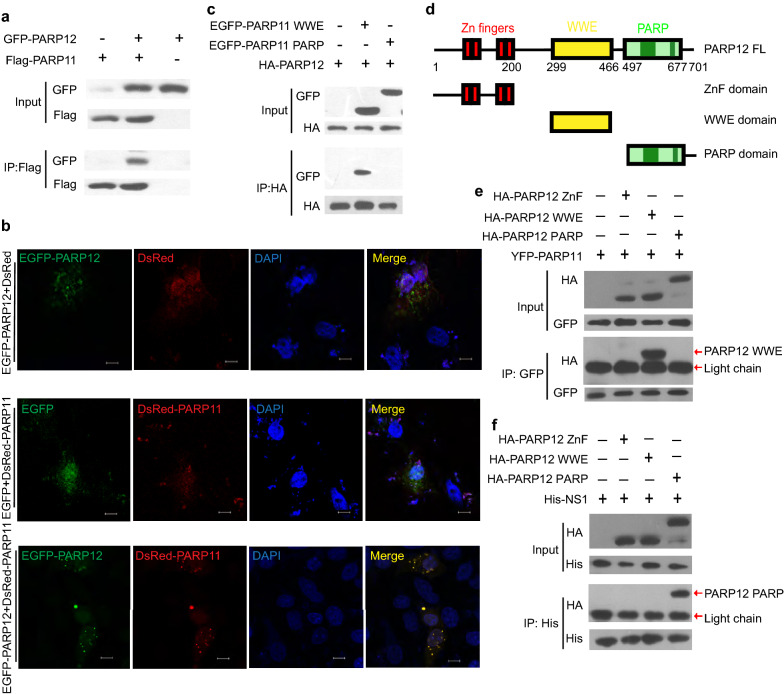
PARP11 interacts and co-localizes with PARP12. **a** Western blotting analysis of lysates immunoprecipitated for PARP12 from HEK293T cells co-transfected with Flag-PARP11 and GFP-PARP12 plasmids. **b** Confocal microscopy analysis of Vero cells that were co-transfected with EGFP-PARP12, RFP-PARP11 and vector control plasmids. Scale bars, 5 μM. **c** Western blotting analysis of lysates immunoprecipitated for PARP12 from HEK293T cells co-transfected with HA-PARP12 and EGFP-PARP11 WWE or EGFP-PARP11 PARP plasmids. **d** Sketch map of the functional regions of full length (FL) PARP12 and mutant constructs. **e** Western blotting analysis of lysates immunoprecipitated for PARP11 from HEK293T cells co-transfected with HA-PARP12 ZnF, HA-PARP12 WWE, HA-PARP12 PARP and YFP-PARP11 plasmids.** f** Western blotting analysis of lysates immunoprecipitated for NS1 from HEK293T cells co-transfected with HA-PARP12 ZnF, HA-PARP12 WWE, HA-PARP12 PARP and His-NS1 plasmids. Western blotting (**a**, **c**, **e** and **f**) and confocal images (**b**) were presentative of three independent experiments

To elucidate the mechanism by which PARP11 suppressed ZIKV in cooperation with PARP12, we firstly examined the interaction between PARP11 and PARP12. We demonstrated that PARP11 could interact and co-localize with PARP12 protein by co-immunoprecipitation and immunofluorescence assay (Fig. 8a, b). To further identify the protein module of PARP11 that interacted with PARP12, we co-transfected HEK293T cells with HA-PARP12 and EGFP-PARP11 WWE and PARP domain expressing plasmids and performed co-immunoprecipitation assay with HA-tagged agarose beads. Full-length PARP12 interacted with the WWE domain of PARP11 but not the PARP domain (Fig. 8c). This result is in line with the function of WWE domain as a common interaction module that participates in both ubiquitination and ADP-ribosylation. We further constructed HA tagged PARP12 mutant which expressed ZnF, WWE and PARP domain of PARP12 (Fig. 8d), and performed immunoprecipitation assay to examine which domain of PARP12 interacted with PARP11. The result indicates that PARP12 interacts with PARP11 through its WWE domain (Fig. 8e). We then performed immunoprecipitation assay to examine which domain of PARP12 interacted with ZIKV NS1 protein, and found that PARP12 interacted with NS1 through its PARP domain (Fig. 8f). These results demonstrate that PARP11 and PARP12 interact and cooperate with each other on ZIKV suppression.

## Discussion

Although the ZIKV outbreak happened in 2015–2017, ZIKV continues to develop and evolve in a form of asymptomatic infectious. According to a previous report, researchers firstly detected the appearance of the ZIKV Africa lineage in Brazil, indicating the risk of a new epidemic [6]. Research on the pathology of ZIKV to explore for available means on epidemic control and associated disease treatment is still urgently needed. In our previously work, we identified PARP12 with anti-ZIKV function and the detailed mechanism was provided [18]. Here, we found that PARP11 suppresses ZIKV in cooperated with PARP12 (Fig. 5a–f). Our studies describe a novel mechanism of PARP11 in viral restriction that is quite different from the previous report indicating that PARP11 promotes VSV and HSV replication by degrading IFNAR1 and inhibiting IFN signaling pathway [26] as we observed no significant change of IFANR1 at protein level in ZIKV infected A549 WT and PARP11-deficency cells (Fig. 3a–d). We speculated the different function of PARP11 may be a result from the characteristic of different virus species.

In this work, we identified *PARP11* as a new anti-ZIKV ISG that suppresses ZIKV replication in cooperation with PARP12. Several PAPRs have been found with anti-viral functions dependent or independent on their PARP enzyme activities. PARP12, for an example, can utilize its PARP enzyme activity to poly ADP-ribosylate ZIKV NS1 and NS3 proteins which promotes their subsequently ubiquitination and degradation by proteasome [18]. PARP13, also known as ZnF antiviral protein (ZAP), inhibits various viruses by directly degrading viral mRNA or protein independent of its PARP enzyme activity [30]. PARP11 was reported as a novel enzyme important for proper sperm head shaping and a potential factor involved in idiopathic mammalian teratozoospermia. In this process PARP11 exhibits mono ADP-ribosylation activity which ADP-ribosylates itself and is essential for co-localization of PARP11 with the nuclear pore components [31]. Other research also indicated that the cellular location of PARP11 is regulated by its PARP catalytic activity [32]. In this work, we found that PARP11 suppresses ZIKV independent on its PARP enzyme activity (Fig. 4a–c) but is still involved in increased protein ADP-ribosylation in ZIKV infected host cells (Fig. 3e, f). We showed that the existence of PARP11 enhances the NS1 and NS3 degradation mediated by PARP12. In addition, the anti-viral function of PARP11 was largely impaired in the absence of PARP12, indicating that PARP11 inhibits ZIKV replication dependent on the existence of PARP12 (Fig. 7a–c). These results suggest that PARP11 cooperates with PARP12 in ZIKV protein degradation through enhancing NS1 and NS3 degradation. We also noticed that PARP11 can suppress ZIKV replication and NS1 and NS3 protein degradation in the absence of PARP12, although at a much lower efficiency than that in the presence of PARP12 (Fig. 7d–g). So, other unrevealed mechanism might be involved in the antiviral activity of PARP11 independent of PARP12. Thus, more attention should be paid to the anti-viral role of PARP family proteins, especially PARP11 and PARP12.

We further identified that the WWE domains of both PARP11 and PARP12 are involved in their interaction (Fig. 8c). Considering that PARP12 ADP-ribosylates NS1 and NS3 dependent on its PARP enzyme activity, PARP12 may work as an intermediate that interacts with NS1 and NS3 proteins by its PARP domain and interacts with PARP11 by its WWE domain. PARP11, NS1 and NS3 proteins, and PARP12 constitute a degradation complex in which PARP11 assists PARP12-mediated ADP-ribosylation in an unknown mechanism.

In conclusion, our work highlights a novel anti-ZIKV role of PARP11 and its mechanism responsible for enhancing PARP-12-mediated NS1 and NS3 degradation. As more and more PARP inhibitors have been developed, further studies on the antiviral activities of different PARP family members will likely provide additional treatments for diseases associated with viruses such as ZIKV.

## Materials and methods

### Virus and cells

ZIKV strain GZ01/2016 (Genbank Accession Number KU820898) and VSV virus was used at a multiplicity on infection (MOI) of 0.1 in this study, except where indicated otherwise [33]. The *IFNAR1*^*−/−*^ HEK293T and A549 cell lines were generated as described [19]. A549, BHK-21, Vero, HeLa and HEK293T cells were purchased from America Type Culture Collection and cultured in Dulbecco’s modified Eagle’s medium (DMEM) (37 °C, 5% CO_2_) supplemented with 10% fetal bovine serum (FBS), 100U/mL penicillin and 50 μg/mL streptomycin.

### Plaque assay

BHK-21 cells were seeded in a 12-well plate for 12 h. Cells were washed with PBS once and infected with virus samples for 1 h. The culture supernatant was aspirated and replaced with DMEM containing 1% low-melting agarose and 2% FBS. Viral plaques were stained and counted 4 days after infection. The titer of ZIKV was quantified by plaque assay and normalized to control.

### DNA constructs and stable cell line generation

pMOI-GFP and pMOI-PARP11 (Homo sapiens) expression plasmids were purchased from GeneCopoeia and described previously [34]. The viral RNA of the GZ01/2016 strain was isolated and used in reverse transcription PCR experiments to obtain the complementary DNA (cDNA) sequence of ZIKV nonstructural NS1 and NS3 proteins. ZIKV NS1 and NS3 genes were cloned into the pcDNA6/V5-His expression vector (Invitrogen) using standard molecular techniques and verified by sequencing. DsRed-PARP11, EGFP-PARP12, Flag-PARP11, HA-PARP12, HA-PARP12 ZnF, HA-PARP12 WWE, HA-PARP12 PARP, EGFP-PARP11 WWE domain, EGFP-PARP11 PARP domain, YFP-PARP11, Flag-PARP13 and GFP-PARP11 mutants were cloned using standard molecular cloning and oligonucleotide mutagenesis methods. To create a stable cell line for PARP11 expression, PARP11 was cloned into the pMXsIG-IgkFLAG vector and co-transfected into HEK293T cells with VSV glycoprotein and pCpG helper plasmids. 48 h after transfection, the culture supernatant was collected and added into WT or *PARP12*^*−/−*^ A549 cells for infection. The cells were collected 72 h after infection, and the PARP11-overexpressing cells were then sorted by fluorescence-activated cell sorting (FACS).

### Western blotting

All cells were treated as indicated and lysed with lysis buffer [50 mM tris–HCl (pH 7.5), 150 mM NaCl, 5 mM EDTA, 1% NP-40, 1 mM PMSF, and 1 × protein inhibitor (Roche)]. The cell extracts were immunoblotted with the indicated antibodies to measure the level of the expressed proteins. Mouse anti-β-actin (ZSGB-Bio), rabbit anti-GFP (Abcam), rabbit anti-PARP11 (Finetest), rabbit anti-IFNAR1(Abcam), mouse anti-poly (ADP-ribose) (GeneTex), rabbit anti ZIKV NS1 (Genetex), rabbit anti-ZIKV NS3 (Genetex), mouse anti-HA, mouse anti-His, and mouse anti-Flag tag antibodies (Sigma-Aldrich) were used for detection at the appreciated dilutions.

### Co-immunoprecipitation assay

HEK293T cells were transfected with the indicated plasmids. 30 h after transfection, protein was extracted using solution A [50 mM tris–HCl (pH 7.5), 150 mM NaCl, 5 mM EDTA, 1% Triton-X100, 1 mM phenylmethylsulfonylfluoride (PMSF), and 1 × protein inhibitor (Roche)]. An aliquot of the extracts was immunoblotted with the indicated antibodies. The remaining extracts were immunoprecipitated using Sepharose beads bound to anti-Flag, anti-HA, anti-His or anti-GFP antibodies (Sigma-Aldrich) at 4 °C overnight. After washing the Sepharose beads four times with solution B [50 mM tris–HCl (pH 7.5), 150 mM NaCl, 5 mM EDTA, 0.2% Triton-X100, and 1 mM PMSF], proteins were eluted by heating the beads to 98 °C in 1 × SDS-polycrylamid gel electrophoresis loading buffer [50 mM Tris–HCl (pH 6.8), 2% (V/V) SDS, 6% (V/V) glycerol, and 2% (V/V) β-mercaptoethanol]. The eluted was analyzed by Western blotting with the indicated antibodies.

### Gene knockout by the CRISPR/Cas9 system

To knockout *PARP11* and *PARP12* in A549 and HEK293T cell lines, two small guide RNAs (SgRNAs) (~ 100 bp gap sequence) targeting the *PARP11* and *PARP12* genes were designed and cloned into sgRNA expression vectors under the control of human U6 promotor. A549 or HEK293T cells were transfected with sgRNAs and Cas9 expression plasmids, followed by puromycin selection, as described previously [35, 36]. Sigle clones were isolated by FACS and confirmed by PCR genotyping and sequencing.

### RNA isolation, reverse transcription, and PCR

Total RNA from cells or viruses was extracted with the PureLink RNA Extraction kit (Thermo Fisher Scientific). Viral RNA copies were measured by qRT-PCR [37] with the One Step PrimeScript RT-PCR kit (Takara). ZIKV primers and TaqMan probes were described previously [38]. Primers used to amplify corresponding genes were obtained from PrimerBank (http://pga.mgh.harvard.edu/primerbank/). SYRB Green qPCR mix (TransGen Biotec) was used to analyze mRNA levels on an ABI 7500 (Applied Biosystems) analyzer.

### Immunofluorescence staining and confocal imaging

Vero cells were seeded in a confocal dish (Solarbio) and transfected with EGFP-PARP12 and DsRed-PARP11 plasmids. After 24 h, cells were fixed with 0.4% paraformaldehyde for 15 min and permeabilized in 0.2% Triton-X100 for 15 min at room temperature. The cells were washed three times with PBS supplemented with 0.05% Tween-20. Nuclei were stained with 4′6-diamidino-2-phenylindole (Thermo Fisher Scientific). Cells were imaged on a LSM700 (Carl Zeiss) confocal microscope, and the images were analyzed with ImageJ software.

### Statistical analysis

All data were analyzed using Prism software (Graphpad 8.0). Statistical evaluation was performed by two-way Student’s *t* test. Data are mean ± SEM, and *P* values are indicated by ns, not significant, *P < 0.05, **P < 0.01, ***P < 0.001 and ****P < 0.0001. All cellular experiments were repeated at least three times.

## Supplementary Information

**Additional file 1: Figure S1.** Construction of *PARP11* knockout and PARP11-overexpressing A549 cell lines. **a** Design of two sgRNA targeting the genome loci of *PARP11* in A549 cells. **b** Deletion of ~ 100 bp genomic DNA in a *PARP11*^*−/−*^ clone was confirmed by PCR. **c** WT and *PARP11*^*−/−*^ cells were immunoblotted for PARP11. **d** Verification of PARP11-overexpressing and vector control A549 (GFP tagged) cells were immunoblotted for GFP. Western blotting results (**b**–**d**) are representative of three independent experiments.

## Data Availability

The datasets used or analyzed during the current study are available from the corresponding author on reasonable request.

## Abbreviations

PARP: Poly-adenosine 5’-diphosphate (ADP)-ribose polymerases
ZIKV: Zika virus
NS1: Non-structural protein 1
NS3: Non-structural protein 3
IFNAR1: IFNα/β receptor subunit 1
VSV: Vesicular stomatitis virus
IFN: Interferon
ISGs: Interferon-stimulated genes
