# Supplementary material for: ADP-ribosyltransferase PARP11 suppresses Zika virus in synergy with PARP12
Source: Cell Biosci. 2021 Jun 29;11:116. doi: 10.1186/s13578-021-00628-y (PMC8240438; doi:10.1186/s13578-021-00628-y)
Supplement: Supplementary file 1 — Additional file 1: Figure S1. Construction of PARP11 knockout and PARP11-overexpressing A549 cell lines. a Design of two sgRNA targeting the genome loci of PARP11 in A549 cells. b Deletion of ~ 100 bp genomic DNA in a PARP11−/− clone was confirmed by PCR. c WT and PARP11−/− cells were immunoblotted for PARP11. d Verification of PARP11-overexpressing and vector control A549 (GFP tagged) cells were immunoblotted for GFP. Western blotting results (b–d) are representative of three independent experiments. [file 13578_2021_628_MOESM1_ESM.docx]

**Supplementary Materials for**

**ADP-ribosyltransferase PARP11 suppresses Zika virus** **in synergy with PARP12**

Lili Li^1,2^, Yueyue Shi^2^, Sirui Li^3^, Junxiao Liu^2^, Shulong Zu^1,2^, Xin Xu^2^, Meiling Gao^1,2^, Nina Sun^4^, Chaohu Pan^2^, Linan Peng^2^, Heng Yang^1,2*^ and Genhong Cheng^5*^

^1^Center for Systems Medicine, Institute of Basic Medical Sciences, Chinese Academy of Medical Sciences & Peking Union Medical College, 100005 Beijing, China

^2^Suzhou Institute of Systems Medicine, 215123 Suzhou, Jiangsu, China

^3^Lineberger Comprehensive Cancer Center, University of North Carolina at Chapel Hill, Chapel Hill, NC 27599, USA

^4^CAS Key laboratory of Infection and Immunity, Institute of Biophysics, Chinese Academy of Sciences, Chaoyang District, Beijing 100101, China

^5^Department of Microbiology, Immunology & Molecular Genetics, University of California, Los Angeles, Los Angeles, CA 90095, USA

HY and GC are senior co-authors of this paper.

Correspondence to: Dr. Heng Yang [yhmyt@hotmail.com](mailto:yhmyt@hotmail.com), and Dr. Genhong Cheng [gcheng@mednet.ucla.edu](mailto:gcheng@mednet.ucla.edu).

**
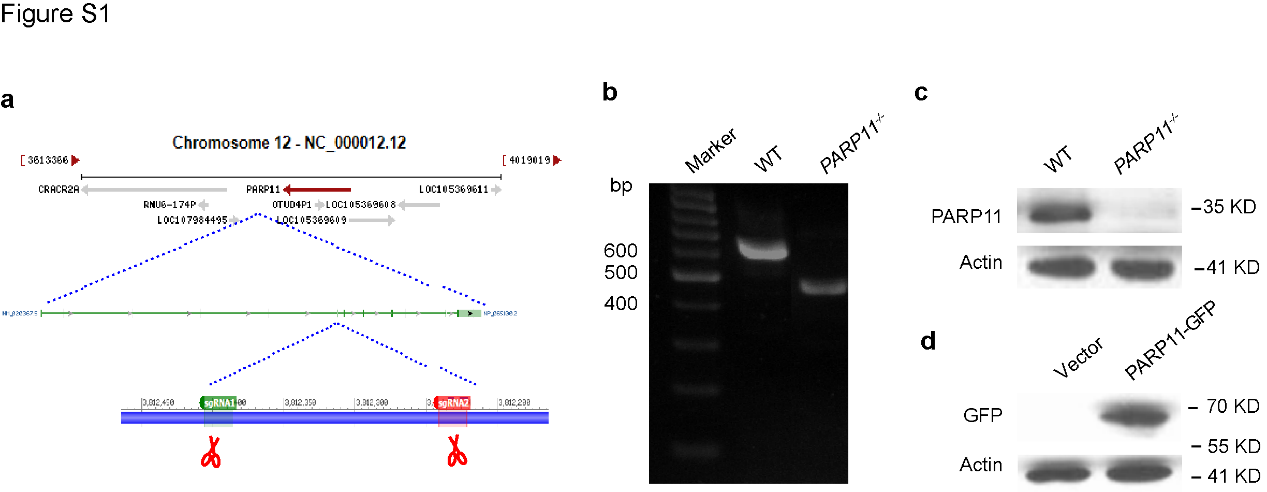
**

**Figure S1. Construction of *PARP11* knockout and PARP11-overexpressing A549 cell lines.** (A) Design of two sgRNA targeting the genome loci of *PARP11* in A549 cells. (B) Deletion of ~100bp genomic DNA in a *PARP11^-/-^* clone was confirmed by PCR. (C) WT and *PARP11^-/-^* cells were immunoblotted for PARP11. (D) Verification of PARP11-overexpressing and vector control A549 (GFP tagged) cells were immunoblotted for GFP. Results (B-D) are representative of three independent experiments.
